# Characteristics of Imperial College London's COVID‐19 research outputs

**DOI:** 10.1002/leap.1358

**Published:** 2021-01-12

**Authors:** Robyn Price, Yusuf Ozkan

**Affiliations:** ^1^ Imperial College London London UK

## Abstract

We identified 651 research outputs on the topic of COVID‐19 in the form of preprint, report, journal article, dataset, and software/code published by Imperial College London authors between January to September 2020. We sought to understand the distribution of outputs over time by output type, peer review status, publisher, and open access status. Search of Scopus, the institutional repositories, Github, and other databases identified relevant research outputs, which were then combined with Unpaywall open access data and manually‐verified associations between preprints and journal articles. Reports were the earliest output to emerge [median: 103 days, interquartile range (IQR): 57.5–129], but journal articles were the most commonly occurring output type over the entire period (60.8%, 396/651). Thirty preprints were identified as connected to a journal article within the set (15.8%, 30/189). A total of 52 publishers were identified, of which 4 publishers account for 59.6% of outputs (388/651). The majority of outputs were available open access through gold, hybrid, or green route (66.1%, 430/651). The presence of exclusively non‐peer reviewed material from January to March suggests that demand could not be met by journals in this period, and the sector supported this with enhanced preprint services for authors. Connections between preprints and published articles suggests that some authors chose to use both dissemination methods and that, as some publishers also serve across both models, traditional distinctions of output types might be changing. The bronze open access cohort brings widespread ‘free’ access but does not ensure true open access.

**Key points**

- All outputs published by Imperial College London authors on the topic of COVID‐19 between January and March 2020 were preprints, reports, and software/code and all published without peer review.
- Across the entire period, January to September 2020, the most common output type from Imperial College London was journal articles, representing 60.8% of all outputs.
- Widespread open access (OA) compliance was observed, with 66.1% of all outputs available as gold, green, or hybrid OA. A further 30.5% of outputs were granted free reading access at the time of reporting under bronze OA. 3.4% of all outputs are closed access.
- 15.8% of the preprints identified resulted in the publication of a journal article in the same time period, with a median of 60 days between publication of the preprint and the journal article.
- Fifty‐two publishers were identified across all outputs, of which 4 publishers account for 59.6% of the outputs.

## INTRODUCTION

The novel coronavirus (SARS‐CoV‐2), the disease it causes (COVID‐19), and its implications for society have been described as the fastest‐moving production of knowledge in our time (Kupferschmidt, 2020) and are estimated to have resulted in tens of thousands of papers produced in a 6‐month period (Teixeira da Silva, Tsigaris, & Erfanmanesh, 2020). As a large, research‐intensive science, technology and medicine university with substantial biomedical and public health expertise, Imperial College London researchers began sharing research on the topic in January 2020, when clinical cases of the disease were limited to China. A group formed under the name ‘Imperial College COVID‐19 Response Team’, comprised of staff from Imperial's MRC Centre for Global Infectious Disease Analysis (MRC GIDA) and the Abdul Latif Jameel Institute for Disease and Emergency Analytics (J‐IDEA), led by Professor Neil Ferguson. With a policy to immediately share research before peer review, the group published epidemiological models, co‐publishing through both the institutional open access repository and their institutional website. The earliest dated output from this group was published on 17 January 2020, 13 days before the World Health Organization (WHO) declared the outbreak to be a Public Health Emergency of International Concern (World Health Organization, 2020b). The forecasts of one report (Ferguson *et al*., 2020) were widely cited as having changed multiple national government responses to the pandemic (Bruce‐Lockhart, Burn‐Murdoch, & Barker, 2020) (Landler & Castle, 2020) (Boseley, 2020). This output received phenomenal media and online attention (https://www.altmetric.com/details/77704842). Many other researchers and groups at Imperial have produced COVID‐19 research in a variety of formats and open access models. We sought to understand the quantity and characteristics of all of Imperial's contributions to COVID‐19 research in order to provide data for the institution to understand its outputs, as well as to provide an institutional cohort perspective to complement the global output level of analysis in other studies on COVID research (Di Girolamo & Meursinge Reynders, 2020; Fraser *et al*., 2020; Helliwell *et al*., 2020; Shuja, Alanazi, Alasmary, & Alashaikh, 2020; Teixeira da Silva *et al*., 2020). The institution's commitment to ‘*consider the value and impact of all research outputs (including datasets and software) in addition to research publications*’ (SF Dora, 2012) as a signatory of the San Francisco Declaration on Research Assessment instructed us to consider the widest possible interpretation of research outputs that were still feasible to collect using bibliographic and data search methods; resulting in journal articles, preprints, reports, datasets, and software/code forming the dataset.

## OBJECTIVES

We sought to understand the volume and characteristics of the research from Imperial College London on the novel coronavirus in a publication period of 1st January to 30th September 2020. The following research aims were identified:

- Identify the volume of publications and the distribution over the time period by different research output types.
- Determine what proportion of preprints went on to be published as journal articles and the average time for this.
- Identify open access trends.
- Demonstrate the distribution of outputs between publishers.

## METHODS

This was a cross‐sectional study of Imperial College London‐authored research outputs related to COVID‐19. The data were extracted in October 2020.

### Search strategy

The search strategy is described in Supplementary data file 1 ‘Search Strategy’. For all steps, the search terms used are ‘2019‐nCoV’, ‘COVID‐19’, ‘SARS‐CoV‐2’, or ‘coronavirus’.

1. Journal articles collected by search performed on Scopus for terms in Title, Abstract or Keywords, and authorship affiliations to ‘Imperial College London’.
2. Reports collected by search performed on Imperial College London's open access publication repository (Spiral) for terms in Title or Keywords and Type as ‘Report’.
3. Datasets collected by search performed on Imperial Research Data Repository for terms in Title and filtered to ‘datasets’. The same search was performed on Zenodo, Google Dataset Search, Open AIRE, and Datacite Search, with these results manually verified to have Imperial authors.
4. Software/code collected by search performed on Imperial College London Github repository, Imperial College London Software Repository, and MRC Centre for Global Infectious Disease Analysis Github repository for terms in Title or About field.
5. Preprints collected by search performed on Imperial College London Current Research Information System (CRIS) for terms in Title, Abstract, or Keywords. Standard Digital Object Identifiers (DOI) prefixes for preprint servers were identified and then used to filter preprint materials in the set.^1^
6. Preprints were also collected through a search on Dimensions for terms in Title and Abstract; ‘Preprint’ in Publication Type and ‘Imperial College London’ in Research Organization. These results were merged with the CRIS preprint results described in Step 5 and deduplicated.
7. All results (excluding arXiv items and software/code without DOIs) were run through the Unpaywall Simple Query Tool to retrieve open access status.
8. Manual update of open access status for arXiv items and software/code.
9. Results were manually screened by exact or similar title and authorship to identify likely preprint/journal article output relationship.

## VARIABLES

### Software definitions

For the equivalent of publication date, the earliest found date in the repository referring to the release or any documented action on the output was taken as a proxy publication date. Anonymous authorship practices in software communities introduce uncertainty around author or affiliated institution. Outputs identified from non‐institutionally managed repositories were manually verified to have Imperial authors before inclusion. Multiple versions of the same software/code published in the same repository file were considered as one entity, dated to their earliest found version.

### Preprint definitions

Multiple versions of the same preprint that shared a common DOI were counted as a single output, but versions with different DOIs or hosted on different servers or repositories were counted as individual outputs. We could not find a systematic way to identify preprints that also existed as journal articles, so we had to identify these connections manually by similarity of title and author composition. We chose to move the contents of the Unpaywall ‘publisher’ field into the ‘journal name’ field for preprints and inputted manually into the ‘publisher’ field the owner of the server, for example, ‘journal name’ becomes ‘medrXiv’ and ‘publisher’ becomes ‘Cold Spring Harbour Laboratory’.

### Peer reviewed and no peer reviewed status

Peer review status was interpreted as *de facto* ‘not peer‐reviewed’ for preprints, reports, datasets, and software/code and *de facto* ‘peer reviewed’ for journal published articles. Exception: Wellcome Open Research and F1000 outputs were considered individually on the basis of their declared peer review status at time of reporting.

### Open access definitions

Open access determination was provided by Unpaywall: closed, bronze, green, hybrid, and gold. Closed access is defined as no access to the relevant output without subscription or log in, including membership log ins for a free item. Bronze access is defined as temporary free access via publishers' websites (to support the global efforts to fight against the pandemic, many publishers provide temporary free access to COVID‐19‐related research outputs). Green open access is defined as items made available by self‐archiving via institutional or subject repositories or preprint servers (excluding preprints published under a CC BY licence). Hybrid open access is defined as an article distributed under CC BY licence in a subscription journal. Gold open access is defined as CC BY (or equivalent distribution for a non‐journal output type) and for an article, in a journal where all content is distributed fully open access (Priem, 2020). We chose to exclude bronze access from the broader open access groups of gold, green, and hybrid with respect to the distinction between ‘free’ access and ‘open’ access that encompasses rights to reuse, revise, redistribute, remix, and retain (Costello, 2019).

## RESULTS

A total of 651 outputs were identified from the search. These included journal articles, preprints, software/code, reports, and datasets. See Table 1 for full details.

**TABLE 1:** Categorization of outputs by type, publisher, and open access status.

		Outputs (*n* = 651)
Type	Journal article	396
	Preprint	189
	Software/Code	29
	Report	29
	Dataset	
Publisher^a^	Cold Spring Harbour Laboratory (medRxiv and bioRxiv)	136
	Elsevier (inc. SSRN)	121
	Wiley (inc Authorea)	75
	Springer	56
	BMJ	31
	Imperial College London	31
	Github	28
	SAGE	18
	Cornell University (arXiv)	15
	Oxford University Press	14
	Research Square	13
	Others^b^	113
Open access status	Gold	282
	Green	78
	Hybrid	70
	Bronze	199
	Closed	22

^a^ Publishers inclusive of all output types; for example, journal publishers, preprint servers, repositories.

^b^ Others *n* < 10 re provided in the Appendix.

### Volume of publication by month

**FIGURE 1:**
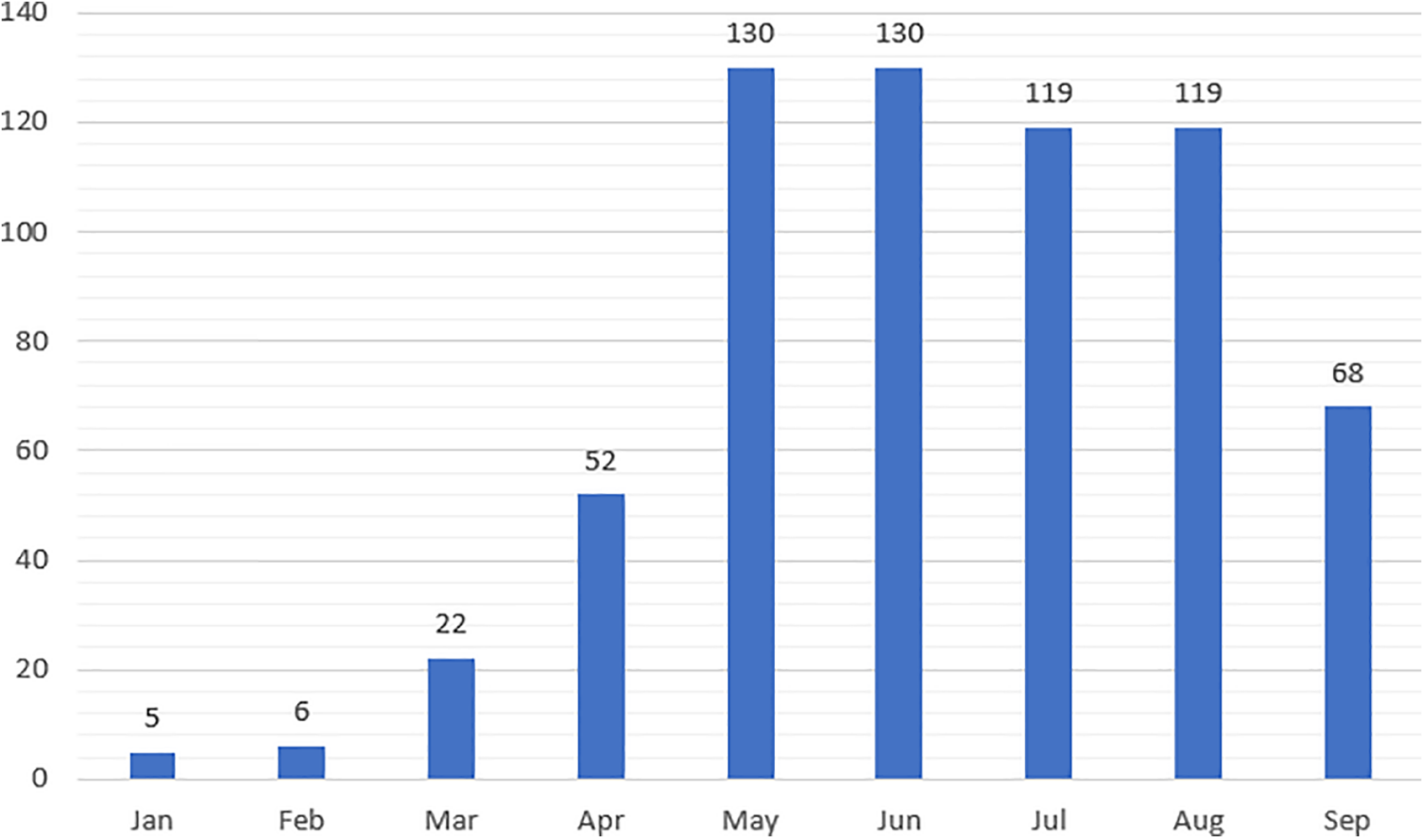
Absolute monthly outputs, all output types.

Month‐on‐month change in the volume of publication was observed across the period, with some instances of no change: January to February (20% growth, 5 vs. 6), February to March (266.7% growth, 6 vs. 22), March to April (136.3% growth, 22 vs. 52), April to May (155% growth, 52 vs. 130), May to June (no change, 130 vs. 130), June to July (−8.4% decline, 130 vs. 119), July to August (no change, 119 vs. 119), and August to September (−42.8% decline, 119 vs. 68) (Fig. 1).

### Days to publication by output type

**FIGURE 2:**
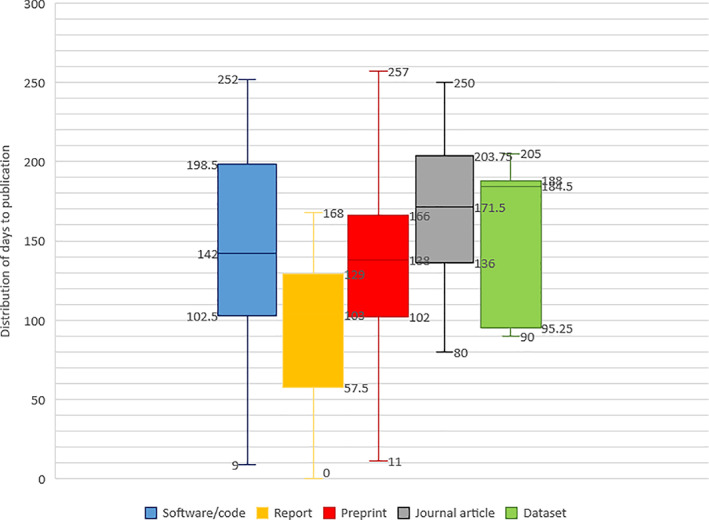
Distribution of publication dates by output type. Day 1 = 17 January 2020 (first publication of an output).

Assuming the first instance of a publication to be Day 1 (report, 17 January 2020) and the final instance of a publication to be Day 257 (preprint, 30 September 2020), we observed the following distribution of publication dates by output type: software/code (median: 142 days, IQR: 102.5–198.5 days), reports (median: 103 days, IQR: 57.5–129 days), preprints (median: 138 days, IQR: 102–166 days), articles (median: 171.5 days, IQR 136–203.75 days), and datasets (median: 184.5 days, IQR: 95.25–188 days) (Fig. 2).

### Peer reviewed and non‐peer reviewed outputs

Across the entire time period, identification of output types as peer reviewed (PR) and non‐peer reviewed (NPR) revealed January to March outputs were exclusively NPR, but across the entire time period, the majority of the outputs were PR (60.8%, 396/651).

**FIGURE 3:**
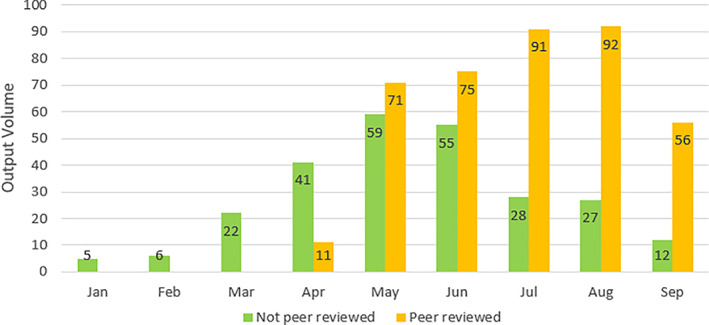
Monthly distribution of peer reviewed versus non‐peer reviewed publications.

- January (NPR 100%, 5/5),
- February (NPR 100%, 6/6),
- March (NPR 100%, 22/22),
- April (NPR 78.8%, 41/52 vs. PR 21.1%, 11/52),
- May (NPR 45%, 59/130 vs. PR 55%, 71/130),
- June (NPR 42.3%, 55/130 vs. PR 57.6%, 75/130),
- July (NPR 23.5%, 28/119 vs. PR 76.4%, 91/119),
- August (NPR 22.6%, 27/119 vs. PR 77.3%, 92/119),
- September (NPR 17.6%, 12/68 vs. PR 82.3%, 56/68) (Fig. 3).

**FIGURE 4:**
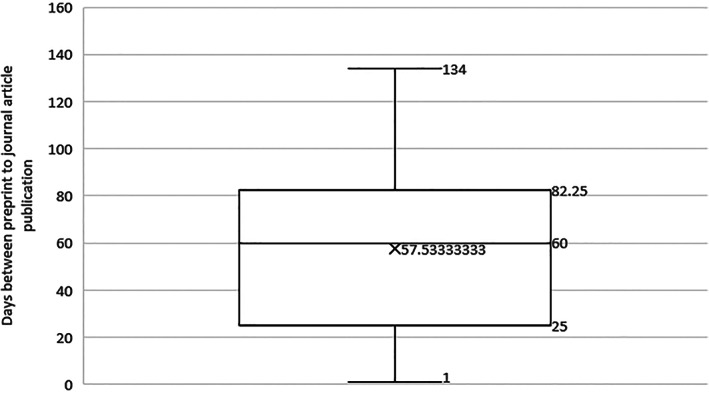
Distribution of days between preprint and journal article publication.

Thirty preprints were identified as later resulting in a journal article publication (15.8%, 30/189). The median time between the preprint publication and the journal article publication was 60 days (IQR: 25–82.25 days) (Fig. 4).

### Diversity of publishers

A total of 52 publishers were identified across the outputs. The top 10 publishers by volume of outputs are:

- Cold Spring Harbour Laboratory (medRxiv and bioRxiv) (20.8%, 136/651);
- Elsevier (18.5%, 121/651);
- Wiley (11.5%, 75/651);
- Springer Nature (8.6%, 56/651);
- Imperial College London (institutional repositories) (4.7%, 31/651);
- BMJ (4.7%, 31/651);
- Github (4.3%, 28/651);
- SAGE (2.7%, 18/651),
- Cornell University (arXiv) (2.3%, 15/651) and
- Oxford University Press (2.1%, 14/651).

**FIGURE 5:**
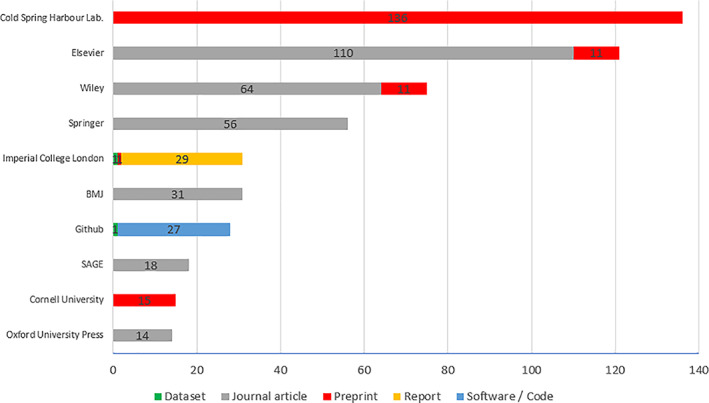
Top 10 publishers by volume of outputs, with output types identified.

Of these 52 publishers, 4 account for 59.6% of the outputs (Cold Spring Harbour Laboratory 21%, Elsevier 19%, Wiley 12%, Springer Nature 9%) (Fig. 5).

### Open access status

**FIGURE 6:**
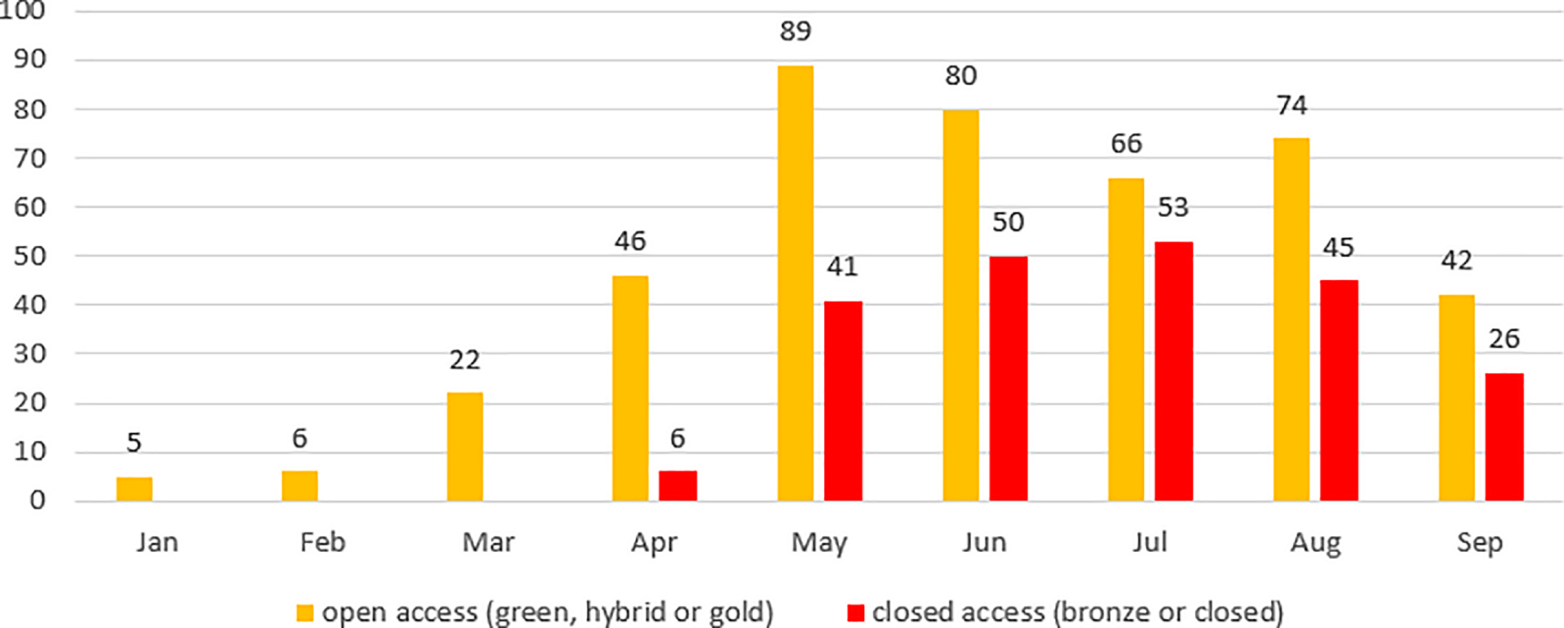
Monthly publication of outputs by open access or closed‐access status.

We identified 66.1% (430/651) of all outputs as open access (gold, hybrid, or green route). The remaining outputs are closed access including bronze (closed access 3.4%, 22/651 and bronze 30.5%, 199/651). In the months January, February, and March, 100% of outputs were published open access, with the introduction of closed‐access outputs from April onwards. Across the entire period, open access items consistently surpass closed‐access publications in monthly output (Fig. 6).

**FIGURE 7:**
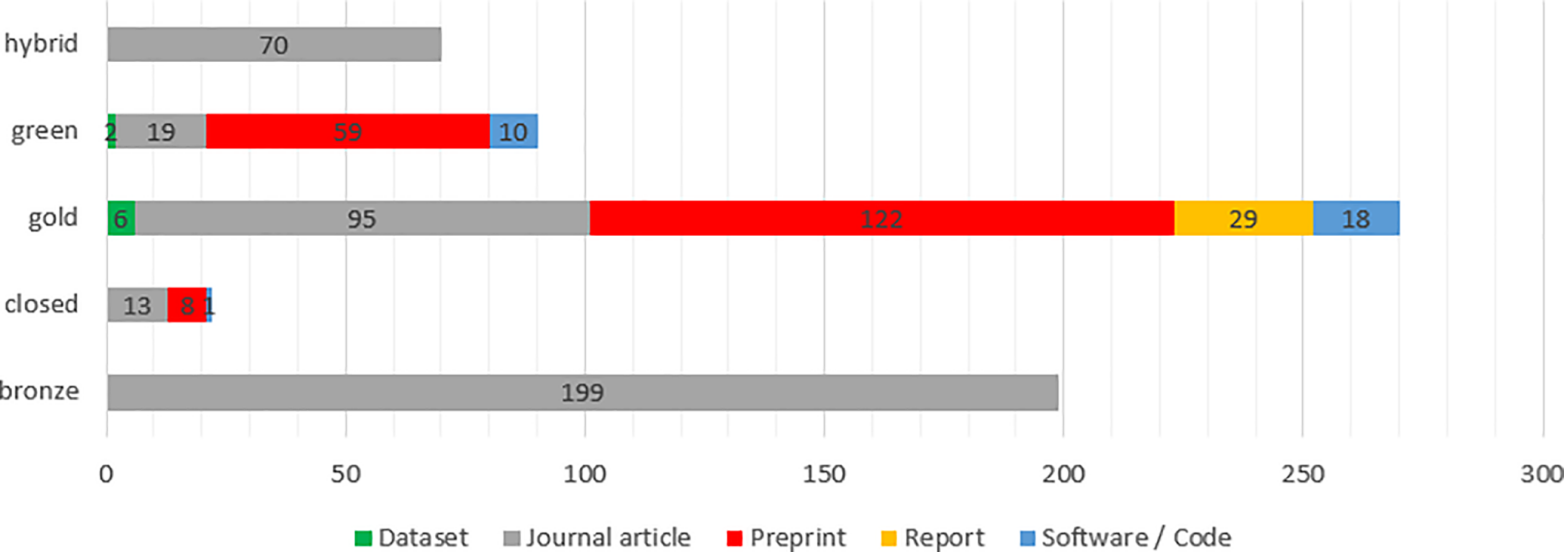
Distribution of OA status by output type.

The following is a breakdown of open access categories by output types:

- hybrid (journal articles 100%, 70/70);
- green (datasets 2.2%, 2/90 vs. journal article 21.1%, 19/90 vs. preprint 65.6%, 59/90 vs. software/code 11.1%, 10/90);
- gold (datasets 2.2%, 6/270 vs. journal articles 35.2%, 95/270 vs. preprints 45.2%, 122/270 vs. reports 10.7%, 29/270 vs. software/code 6.7%, 18/270);
- closed (journal articles 59.1%, 13/22 vs. preprints 36.4%, 8/22 vs. software/code 4.5%, 1/22);
- bronze (journal articles 100%, 199/199) (Fig. 7).

**FIGURE 8:**
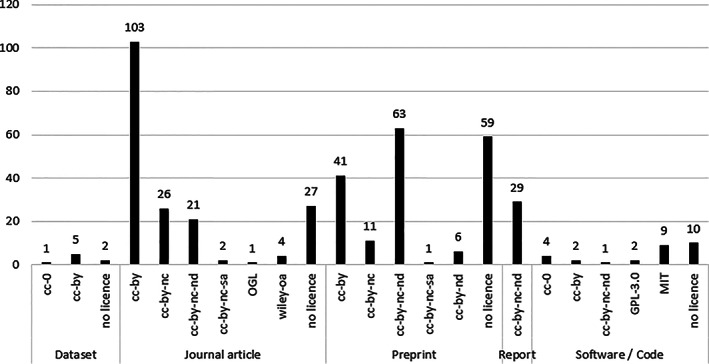
Open access licence breakdown by output type. Note that due to licensing data irregularities, licence does not correspond directly to OA status. Bronze and closed‐access outputs excluded.

Creative Commons licences were observed across journal articles (38%, 152/396), preprints (65%, 122/189), software/code (17%, 5/29), reports (100%, 29/29), and datasets (75%, 6/8). Across all output types, the most popular variation of the Creative Commons licence used was CC BY, the least restrictive Creative Commons licence and used by 151 outputs overall (23%, 151/651). Other open access licences used are broadly permissive: Wiley OpenOnline, Open Government Licence, MIT License, and GNU General Public License (Fig. 8). However, for 22.8% (98/430) of the open access outputs (green, gold, and hybrid routes), an open access licence was not identified. This suggests that either a licence existed but, through a data or file error, it could not be found by our searches and Unpaywall, or that an open access licence does not exist for the item.

## DISCUSSION

Although the majority of outputs over the entire time period were journal articles, the exclusive presence of NPR outputs reports and preprints between January and March, which were not surpassed by PR content until May, suggests that authors needed a faster form of dissemination than journals could offer in the early months of coronavirus pandemic (Kupferschmidt, 2020), similar to those working in other global health emergencies (Zhang, Zhao, Sun, Huang, & Glänzel, 2020). As authors chose to disseminate research in the preprint form, the sector responded. PubMed Central adapted to include coronavirus preprints (www.ncbi.nlm.nih.gov/pmc/about/nihpreprints/), and other existing preprint servers have adapted to prioritize this research or have been established solely for the crisis (Lu Wang *et al*., 2020). Journal publishers responded to the crisis; a decrease of days between submission and publication by some medical journals publishing on the topic has been observed (Horbach, 2020), as well as announcements of reduction of peer review times by publishers (Redhead, 2020). However, whether the likely contradictory demands of both reducing peer review and editorial time whilst retaining quality (Kwon, 2020) are sustainable or achievable are yet to be evaluated long term. There is some indication that this pressure is changing journal publisher attitudes to preprints, seen by explicit encouragement of preprints on the topic at The New England Journal of Medicine (Rubin, Baden, Morrissey, & Campion, 2020), reference to the pandemic as a reason for The Lancet's decision to make their ‘Preprints with the Lancet’ SSRN platform permanent in September 2020 (Kleinert & Horton, 2020), and the introduction of a default preprint policy for COVID‐19 submissions at eLife (Eisen, Akhmanova, Behrens, & Weigel, 2020). Publication platforms such as Wellcome Open Research and F1000 further disrupt traditional distinctions in the journal and peer review process.

As preprints shift closer to the centre field of established scholarly communications, either the infrastructure and data standards supporting them needs to develop, or bibliographic tools need to adapt to accommodate. The complicated method of preprint data collection in this study (searching through the institution's CRIS records, a search function only available to administrators at the institution, and then supplementing it with a second search on Dimensions) was used because, although some databases index preprints (Europe PMC, Dimensions), contributor affiliation data associated with preprints is not of sufficient quality or sufficiently widespread to enable comprehensive search with verified affiliation. This is not a fault of the databases but perhaps a dependency on structured and parsable metadata from preprint servers that is not always available. Also, a lack of accessible methods through which to search for connected preprints and published journal articles, also perhaps due to missing associated metadata identifiers; prevents large‐scale or automated data collection and requires associations to be identified manually as in this study. This constraint could be possibly prohibiting the rich insight that could come from easily accessible mapping of preprint and article networks.

The presence of 52 publishers found is an indication that authors are served with competitive options from which to choose their own preferred outlet for dissemination and are safeguarded against ‘lock‐in’ from any one provider. Whilst the majority of publishers predominantly serve one output type, e.g. journal publishers to journal articles, some are represented across more than one type – for example, the institutional repository publishing as ‘Imperial College London’ is represented amongst datasets (1), preprints (1), and reports (29). This could be a positive indicator that artificial distinctions in the research life cycle are being replaced with more holistic solutions that offer dissemination for all outputs of research. However, others have raised concern that the representation of commercial publishers across output types poses a threat to equity and value in the research production cycle (Posada & Chen, 2018). The acquisition of preprint servers by commercial publishers, Elsevier and SSRN (2016) and Wiley and Authorea Inc. (2018), contributed to their combined preprint and journal article shares in our set (Elsevier 19% and Wiley 12%).

That 100% of papers were published open access in the first 3 months of the pandemic suggests an author preference for this model in this period. However, considering that all of these outputs were not peer reviewed (NPR) types (preprints, reports, and software/code), it is difficult to robustly argue that these outputs were open access as a conscious choice and not a consequence of the NPR output type. There are examples in the full time period of NPR outputs published closed access (the SSRN preprints considered closed due to their membership log in wall and one item of software set to internal view (private) in the Imperial Github repository) across the entire time period, but their presence is small (1.4%, 9/651). Publisher intervention to convert content to bronze open access is positive but has limitations; the access is not ensured in perpetuity and could be revoked in the future (Elsevier, 2020), and conditions of rights are not consistently clarified. Areas of particular need in this crisis that free access alone does not ensure are machine access for text and data‐mining purposes, which is needed to apply artificial intelligence and machine‐learning techniques to COVID‐19 research (Shuja *et al*., 2020) and translation rights to disseminate in a global public health event.

This study of a single institution's outputs was undertaken with an awareness that Imperial is not the largest contributor by publication volume to COVID‐19 research (Hook & Porter, 2020) and obviously not the only institution to have produced impactful results. Despite suggestions of the pressures of adapting research practices to accommodate lab closures and the demand for rapid results leading to smaller teams and fewer international collaborative partners in the early months of the pandemic (Fry, Cai, Zhang, & Wagner, 2020), we understand that coronavirus research demands collaboration at every level (Apuzzo & Kirkpatrick, 2020) and that any institutional‐level analysis should be interpretated in relation to organisation size, mission and resouces.

## LIMITATIONS

We recognize the limitations of comparing output types without adjusting for their characteristics or context. For example, comparison of publication times of journal articles and preprints is not a truly fair comparison given the vastly different time enterprises required of each type; neither is to compare the open access models of output types that are mandated for open access (e.g. articles) and the other output types which are not (preprints, datasets, reports, software/code).

The green open access share of the data may underrepresent the true number of articles self‐archived, an action that is mandated by the institution's open access policy. This is because outputs would only be classified green when there is no publisher‐hosted option available (Piwowar *et al*., 2018), so it is possible that some of the bronze open access items also exist as repository‐archived green open access, but the Unpaywall hierarchy gives authority to the bronze publisher‐hosted version in classification.

## CONCLUSION

Authors were served with options to publish rapidly in non‐peer review form and under open access models throughout the entire period, and from January to March, these options were exclusively used. Across the entire period, however, the most commonly observed output was journal articles. The association of some preprints with journal articles suggests that the status of peer review versus non‐peer review is, for some outputs, not binary. This increasing connectedness between the two can also be seen by the presence of publishers serving across both types. That the majority of outputs were published under some form of open access is positive; however, whether the bronze OA cohort is truly compliant with the long‐term needs of this global challenge (World Health Organization, 2020a) (Wellcome, 2020) is not clear. The inclusion of reports, preprints, datasets and software/code as output types permits a richer and more accurate description of the institution's activities and talents than considering journal articles alone. There is a need for bibliographic methods to adapt to better identify and classify these valuable non‐journal output types.

## CONFLICT OF INTEREST

Both authors declare that they are employees of Imperial College London, UK.

## Supporting information


**File S1.** Supplementary Information.

## Data Availability

Data collected for this study are available at https://doi.org/10.5281/zenodo.4269922.

## Footnotes

a A ‘CRIS’ is the database used by a research organization to manage data relating to the organization's research outputs. Imperial College London's CRIS is Symplectic Elements: www.symplectic.co.uk/
b. The following prefixes were identified: medRxiv and bioRxiv: 10.1101; SSRN: 10.2139; Wellcome Open Research: 10.12688; Research Square: 10.21203; and Authorea: 10.22541. arXiv does not assign DOIs.

## Appendix

**Table:**

Publisher <10	Outputs (*n* = 651)
Informa UK Limited	9
Ovid Technologies (Wolters Kluwer Health)	8
Zenodo	7
Wellcome Trust	7
JMIR Publications Inc.	6
MDPI AG	6
American Association for the Advancement of Science (AAAS)	5
American Thoracic Society	5
European Respiratory Society (ERS)	5
Public Library of Science (PLoS)	4
American Medical Association (AMA)	4
S. Karger AG	4
Frontiers Media SA	4
Mary Ann Liebert Inc	3
Mark Allen Group	2
EMBO	2
Massachusetts Medical Society	2
American Society of Nephrology (ASN)	2
American Chemical Society (ACS)	2
Institute of Electrical and Electronics Engineers (IEEE)	2
European Centre for Disease Control and Prevention (ECDC)	2
American College of Physicians	2
Royal College of General Practitioners	2
Joule Inc.	1
Medknow	1
World Health Organization Regional Office for the Eastern Mediterranean (WHO/EMRO)	1
Microbiology Society	1
Asociatia Pentru Cresterea Vizibilitatii Cercetarii Stiintifice (ACVCS)	1
Radiological Society of North America (RSNA)	1
Dryad	1
Royal College of Physicians	1
FapUNIFESP (SciELO)	1
Royal Society of Chemistry (RSC)	1
American Physiological Society	1
Sociedade Brasileira de Cardiologia	1
E.U. European Publishing	1
The Royal Society	1
Cambridge University Press (CUP)	1
Ubiquity Press, Ltd.	1
International Union Against Tuberculosis and Lung Disease	1
Walter de Gruyter GmbH	1
