# Supplementary material for: Characteristics of Imperial College London's COVID‐19 research outputs
Source: Learn Publ. 2021 Jan 12;34(3):358–69. doi: 10.1002/leap.1358 (PMC8014869; doi:10.1002/leap.1358)
Supplement: Supplementary file 1 — File S1. Supplementary Information. [file LEAP-34-358-s001.docx]

# Characteristics of Imperial College London’s COVID-19 research outputs

# Supplementary data file 1 “Search Strategy”

Search strategy for retrieving Imperial COVID-19 outputs

**Journal Articles**

Scopus search

 TITLE-ABS-KEY ( "2019-nCoV" )  OR  TITLE-ABS-KEY ( "COVID-19" )  OR  TITLE-ABS-KEY ( "SARS-CoV-2" )  OR  TITLE-ABS-KEY ( "coronavirus" )  AND  AF-ID ( "Imperial College London"   60015150 )

= 449 results

Results screened for:

1. Duplicate publications
2. Outside of Jan 1^st^ – Sep 30^th^ 2020 publication window
3. No online publication date
4. No DOI

= 396 results

**Reports**

Imperial College London open access publication repository search:

"2019-nCoV" "COVID-19" "SARS-CoV-2" "coronavirus" AND type: “reports”

= 29 results

**Datasets**

Imperial College London Research Data Repository search:

"2019-nCoV" "COVID-19" "SARS-CoV-2" or "coronavirus" in title and “dataset” in type

= 8 results

Zenodo, Google Dataset Search, Open AIRE and Datacite Search search:

1. "2019-nCoV" "COVID-19" "SARS-CoV-2" or "coronavirus" in title AND manual verification of Imperial College London authors
2. Deduplicate against results from prior search

= 8 results

**Preprints**

Search for "2019-nCoV" "COVID-19" "SARS-CoV-2" or "coronavirus" in title of Imperial College London Current Information System (CRIS).

= 4597 results

Results screened for:

1. Preprint type identified by DOI prefixes of preprint servers, ‘Type’ and ‘Data Source’ fields
2. Duplicate publications merged
3. Jan 1^st^ – Sep 30^th^ 2020 publication window

= 111 results

Dimensions search:

Search for "2019-nCoV" OR "COVID-19" OR "SARS-CoV-2" OR "coronavirus” AND RESEARCH ORGANIZATION “Imperial College London”. Filter Publication Type to “Preprint”.

= 115 results

Remove 1 publication for out of scope

=114 results

Combine and deduplicate results from CRIS and Dimensions search

= 189 results

**Software/code**

Search Imperial College London Software Repository, Imperial College London Github repository and MRC Centre for Global Infectious Disease Analysis Github repository:

"2019-nCoV" "COVID-19" "SARS-CoV-2" or "coronavirus" in Title or About.

Deduplicate results from both sources.

= 29 results
